# Concussion of the Brain

**Published:** 1875-07

**Authors:** J. D. Rush

**Affiliations:** Alabama


### CONCUSSION OF THE BRAIN—VENESECTION.

BY J. D. RUSH, M.D., OF ALABAMA.

On the 17th of March, 1875, I was called to see a woman (colored) five miles from town, who, on resting herself against the railing around a gallery, it gave way, letting her fall some five feet. Her feet caught on the end of the floor, bringing her head in contact with the sill of the house, producing concussion of the brain. I suppose it was two hours from time of accident until I saw her. I found her in a convulsed state, with singultus, pulse small and intermittent, head thrown back, with oscillation of the eyes, and occasional wide stare and low, muttering voice. Finding her in this condition, and it being in the night, I made but a very partial examination until she was put under the influence of chloroform, when I investigated the case more closely. Finding her to be a very muscular and plethoric woman, I resorted to venesection, taking about sixteen oz. of blood through this means, and obtained complete relaxation of the system. I then gave stimulants until the circulation was fully equalized. I kept her under the influence of chloroform several hours, as she seemed to be suffering very much. After this I applied a large blister of the cerate of flies to the back of the head, letting it extend down the neck several inches. I then put her on full doses of bromide potass. and morphine sulphas, every two hours, sufficient to allay all pain and nervous excitement. She remained in an unconscious state twenty-four hours, from which time she began to rally and continued to improve, and is now able to do very good work.

---

*Local Treatment of Diphtheria.*—Dr. J. Lewis Smith (*New York Medical Record*) reccommends the following local application to the fauces every three hours in cases of diphtheria. To five drops of carbolic acid are added two drachms of subsulphate of iron and one ounce of glycerin. A brush is used in making the application, as less painful, and less liable to produce bleeding. This application, in his hands, has been more satisfactory than any other.—*Detroit Review.*

